# Feasibility of a live-stream group dance intervention with inpatients in subacute post-stroke rehabilitation: A pilot study

**DOI:** 10.1177/20552076261459521

**Published:** 2026-06-05

**Authors:** Lucie Beaudry, Corinne Skaff, Simon Brière, Dominic Létourneau, Michel Tousignant, François Michaud

**Affiliations:** 1 Dance Department, 14845 Université du Québec à Montréal, Québec, Canada; 2 Institut universitaire sur la réadaptation en déficience physique de Montréal, 459878 Centre for Interdisciplinary Research in Rehabilitation (CRIR) of Greater Montreal, Montréal, Québec, Canada; 3 Research Center on Aging (CDRV), 7321 Université de Sherbrooke, Québec, Canada; 4 Department of Electrical Engineering and Computer Engineering, 7321 Université de Sherbrooke, Québec, Canada; 5 Interdisciplinary Institute for Technological Innovation (3IT), 7321 Université de Sherbrooke, Québec, Canada; 6 School of Rehabilitation, 7321 Université de Sherbrooke, Québec, Canada

**Keywords:** telerehabilitation, online/live-stream intervention, dance-based intervention, stroke, rehabilitation, subacute phase, opentera telehealth software platform, telehealth

## Abstract

**Background:**

Stroke rehabilitation settings are increasingly seeking to add complementary therapies to disciplinary interventions to intensify and optimize post-stroke care, particularly during the first three months following the stroke. Dance-based interventions have been studied in this context, but the challenges of space availability in rehabilitation settings can hinder such interventions. As telerehabilitation services become more widespread, it is worth considering whether dance could be offered online in patients’ rooms.

**Purpose:**

We sought to co-develop a live-stream group dance intervention with clinicians and test its feasibility in subacute post-stroke rehabilitation.

**Methods:**

This two-part qualitative study combined collaborative research with a pilot study. Clinicians (n=7), patients (n=6) and personal support workers/PSWs (n=5) were recruited. The patients were undergoing subacute rehabilitation post-stroke (≤21 days) and were recruited using inclusive criteria. Data were collected through focus groups (n=3) with clinicians, video recordings of dance sessions, a research journal, and semi-structured interviews with patients and PSWs. Data analysis combined analytical questioning and thematic analysis.

**Results:**

After identifying safety concerns, dance content was developed for six movement categories, including “body awareness”, “breathing”, and “stretching”. Technical factors (Internet and software connection issues) and environmental factors (ambient noises and sound of music) significantly hindered the intervention. The use of high-gain wireless USB adapters and noise-cancelling headphones were the main solutions applied to improve the intervention and limit the need for PSW assistance.

**Conclusions:**

A live-stream group dance intervention is feasible for adding therapy hours in subacute post-stroke rehabilitation but requires controlling environmental factors and ensuring favourable technical conditions.

## Introduction

A stroke is a sudden loss of brain function caused by an interruption in blood flow (ischemic stroke) or a rupture of a blood vessel (hemorrhagic stroke) inside the brain.^1^ In both cases, stroke causes sudden and massive death of brain cells in the affected area, which can lead to various sequelae. These sequelae can, among other things, affect organic functions such as energy and drives (including motivation), attention, memory, emotions, perception, language, proprioception, pain, exercise tolerance (including fatigue), joint mobility, muscles, control of voluntary movements, or gait.^2^ To recover from these aftereffects, stroke rehabilitation relies on several disciplinary approaches that are commonly used as needed. During subacute post-stroke rehabilitation, which occurs within three months of the stroke, the minimum intensity recommended to optimize patient rehabilitation is three hours per day, five days per week.^3^ In reality, however, rehabilitation generally remains insufficient,^4^ even though hospitalized patients want more intensive rehabilitation.^5^ Some argue that increased activity during leisure time could contribute to improved rehabilitation.^6^ Likewise, rehabilitation settings are increasingly seeking to add complementary and alternative therapies to disciplinary interventions to further optimize post-stroke care.^7,8^

Dance-based interventions have already been researched in stroke rehabilitation, including subacute post-stroke rehabilitation.^9–12^ Usually offered as in-person group classes, they have provided a promising way to complement stroke rehabilitation by delivering physical, psychological, cognitive, and social benefits likely to foster neuroplasticity.^13^ However, lack of space and resources in clinical settings can hinder implementation of such interventions.^14^ While dance-based exercise video games (exergaming) have also been used in the chronic phase of stroke (≥6 months after the stroke),^15–18^ this is not the case in the subacute phase, and dance via telerehabilitation still appears relatively unexplored for people with stroke.

Lee et al.^19^ conducted a randomized controlled trial (feasibility study) on the effects of dance therapy using telerehabilitation in hemiplegic patients in the subacute and chronic phases of stroke (within one to 24 months of onset). They recruited 17 participants (nine in the experimental group and eight in the control group), with inclusion criteria being hemiplegia occurring more than one month after the first stroke, as well as the ability to maintain a sitting position, walk 10 metres independently (or with minimal assistance), and tolerate 40 minutes of activity. The experimental group received dance-therapy sessions in addition to conventional physical therapy, while the control group received only physical therapy. The dance therapy consisted of 40-minute one-on-one sessions, twice weekly for three weeks, using the videoconferencing platform Zoom (Zoom Video Communications Inc. San Jose, CA). Offered in an independent space resembling a home-based rehabilitation setting, their dance-therapy program focused on trunk control and balance training. Their results showed that their remote dance-therapy program was safe and may have similar effects to conventional treatment in terms of improving trunk control in stroke patients, while also improving movement in daily life and balance function.

Lee et al.’s findings echo those of other studies on telerehabilitation after stroke, which confirm its feasibility, usability, and acceptability for ensuring continuity of care and improving access to rehabilitation therapies for stroke patients.^20–22^ In recent years, various telerehabilitation proposals for stroke have emerged, notably thanks to a national initiative supported in Canada by the Heart and Stroke Foundation.^23^ Six major Canadian clinical trials funded by this initiative have shown that telerehabilitation is as effective as traditional face-to-face rehabilitation and that patients are satisfied with this type of service when it is appropriate and involves some social interaction.^24^

Inspired by Lee et al., we sought to go a step further by validating the feasibility of offering a live-stream group dance intervention to hospitalized patients in the subacute phase of rehabilitation, using more inclusive selection criteria and bringing dance into patients’ rooms. The goal was to use dance to increase the number of hours of motor and cognitive stimulation for these patients, while often dealing with limited access to the facilities needed for dance-based interventions due to lack of availability. A complementary virtual intervention could mitigate this problem and supplement rehabilitation services, in addition to potentially paving the way for a live-stream dance service after clinical rehabilitation, as people with stroke often have few opportunities to continue exercising at home.^25^ Prior to the feasibility study, we also sought to develop dance content that could be safely performed remotely (online) while meeting the needs experienced by the patients in this phase of stroke recovery.

## Method

This qualitative study comprised two intertwined parts grounded in two research paradigms. The first paradigm was constructivist, as the study sought to co-develop the content of a live-stream group dance intervention based on collaborative research.^26^ This co-development aimed to integrate scientific and empirical knowledge from the fields of dance, stroke rehabilitation, and telerehabilitation. The second paradigm was pragmatic, as co-developing the intervention made it possible to conduct a pilot study^27^ of the research-experimentation type^28^ aimed at testing the intervention’s feasibility and resolving any potential problems. This six-month study was conducted and reported in accordance with the Consolidated criteria for Reporting Qualitative Research Guidelines^29^ to ensure the quality of the study’s execution and reporting (see Table S1).

### Researcher team characteristics

The lead researcher (PhD) has training in dance and somatics (gentle movement approaches based on body awareness), as well as extensive experience in dance for post-stroke rehabilitation. She also has interdisciplinary experience in health and has previously collaborated with the rehabilitation hospital team that partnered on this study. Even though she had not yet developed live-stream interventions for post-stroke rehabilitation, she had made such adaptations for other intervention contexts. The research assistant also has training in dance and somatics, and extensive experience teaching dance in various contexts, but not specifically to people with stroke. Therefore, she was supervised for this study. Their pooled theoretical and practical knowledge of dance and somatics was combined with the interdisciplinary perspectives of clinicians working in subacute post-stroke rehabilitation, and with the expertise of the co-researchers in telehealth and telerehabilitation (more specifically, for the use of the OpenTera telehealth software platform). These co-researchers were all men (two professors/PhDs, and two software engineers/MAScs).

### Context

The study was conducted in a rehabilitation hospital with a neurology program offering intensive functional rehabilitation services for stroke patients, which include the repetition of functional movements to stimulate brain plasticity and promote the creation of new neural connections following a stroke. This hospital adheres to the Planetree approach,^30^ which advocates, among other things, a path through the arts and an openness to complementary therapies.^31^

### Participants and recruitment

The study included three categories of participants to be recruited using convenience sampling: clinicians, patients, and personal support workers (PSWs). The participating clinicians were therapists specialized in post-stroke rehabilitation within the neurology program of the rehabilitation hospital hosting the study. Working within this program was the sole selection criterion. Prior to the feasibility study, their role was to enhance the relevance and safety of the live-stream group dance intervention through the process of co-developing dance content. For the feasibility study, the participating patients were adults who had recently suffered a stroke (≤21 days) and were undergoing intensive functional rehabilitation within the neurology program. All were eligible for inclusion, regardless of their stroke type and neurological impairments. However, they had to be able to maintain balance while seated, communicate verbally, and understand verbal instructions. Patients with visual impairments that could hinder their participation in a live-stream intervention were excluded, as were aggressive patients with known symptoms of dementia or Alzheimer’s disease prior to their stroke. We did not exclude any potential participants on the basis of fatigue, given that it was possible to skip a session if they were too tired, or to take breaks during the sessions if necessary. Once recruited, each participating patient was assigned a therapist (physical therapist) who would respond in case of emergency. All patients had to have sufficient cognitive abilities to give informed consent and participate in interviews. Like the participating clinicians, the PSWs were employees of the rehabilitation hospital. To be eligible for recruitment, they had to work with patients undergoing stroke rehabilitation. Their role in the feasibility study involved setting up the telehealth equipment in the participating patients’ rooms and collecting it after the sessions for storage, assisting patients if needed during the live-stream dance sessions, and providing follow-ups with the dance professionals when necessary. At the time of recruitment, all participants were given an explanation of their role and the study’s objectives.

Clinicians were approached by the hospital’s neurological care coordinator to gauge their interest in participating in the study. Their professional email addresses and telephone numbers were then forwarded to the research team for recruitment purposes. The same coordinator performed patient screening, while the research team performed patient recruitment. Six patients were sought to create two groups of three, i.e., small groups to ensure the safety of the live-stream dance intervention (as heterogeneous cases were to be expected within each group). The study’s highly exploratory nature, the many unknowns in the field, and especially the limited number of portable telehealth kits at our disposal dictated the small sample size of participating patients. Finally, the coordinator selected PSWs interested in participating in the study and provided the research team with their telephone numbers. A few extra were recruited to fill in when any given PSW could not attend a session. All participation in the project was voluntary. The study received prior approval from the Human Research Ethics Committee of the Université du Québec à Montréal (4893_e_2021). As a private institution under contract with the provincial government, the partner site hosting the study did not have an ethics committee as such.

### Intervention

The live-stream group dance intervention lasted 60 minutes per session, twice a week for four weeks, for a total of up to eight sessions. The sessions involved a variety of movements accompanied by music. The basic structure of the sessions included a welcome ritual (∼3 min), a warm-up (∼10 min), an exploration/development segment (∼25 min), a creative/expressive segment (∼10 min), a cool-down (∼10 min), and a closure ritual (∼2 min). The dance content developed for these different segments is described below as research findings. All movements could be performed with technical aids or adapted depending on each patient’s impairment.

**Figure 1.**
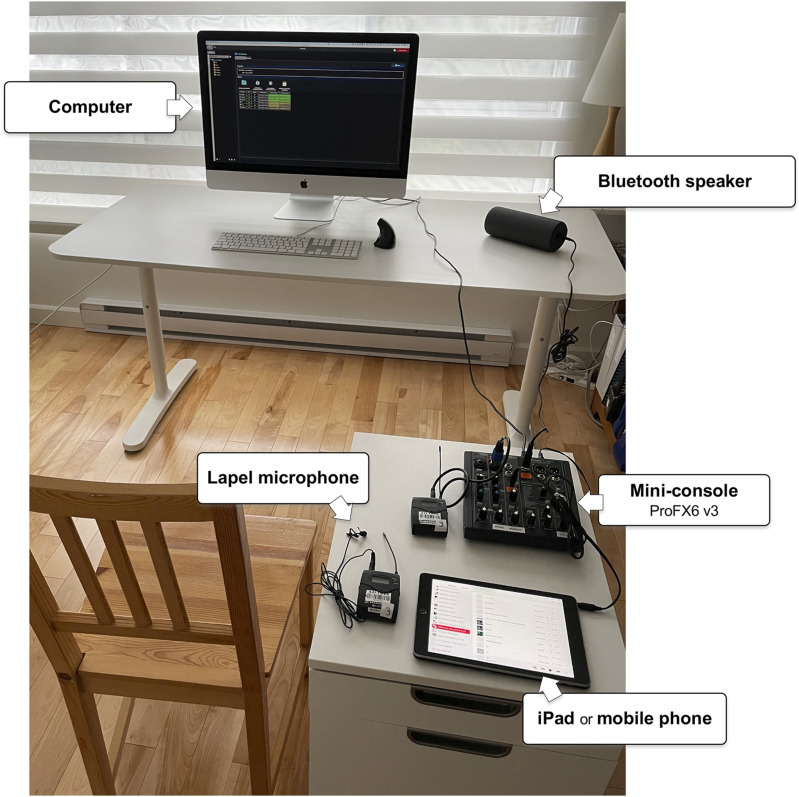
Live-stream dance teaching setup for the dance professional.

The sessions were led by two dance professionals (women), one per group of three patients (due to scheduling availability). The first dance professional was the research assistant, while the second was the lead researcher. This live-stream intervention was conducted using the open source (Apache License 2.0) OpenTera telehealth software platform,^32^ which was designed to offer telerehabilitation sessions and support research projects. At the time of the study, OpenTera did not allow music to be shared via the computer, so we had to find a workaround to ensure a good voice-music mix (this feature has since been developed). A lapel microphone was used to capture the voice of the dance professional, while the music came from an iPad or a mobile phone (see Figure 1), both of which were plugged into a mini mixing console. The music could be heard by the dance professional via a Bluetooth speaker, also plugged into the console. The speaker was placed at some distance from the lapel microphone to prevent feedback.

The dance professionals used a specially tailored desktop application called OpenTera+. OpenTera+ allowed them to connect from their workspace to the platform and start their group session, and each patient in the group to join from their hospital room. The patient (or the PSW) authorized the communication with the dance professional and other patients taking part in the session by tapping on a simple green button on the touchscreen. By forming groups of three patients, the on-screen display was optimized as it enabled each person to properly see the others, with a larger thumbnail for the dance professional.

**Figure 2.**
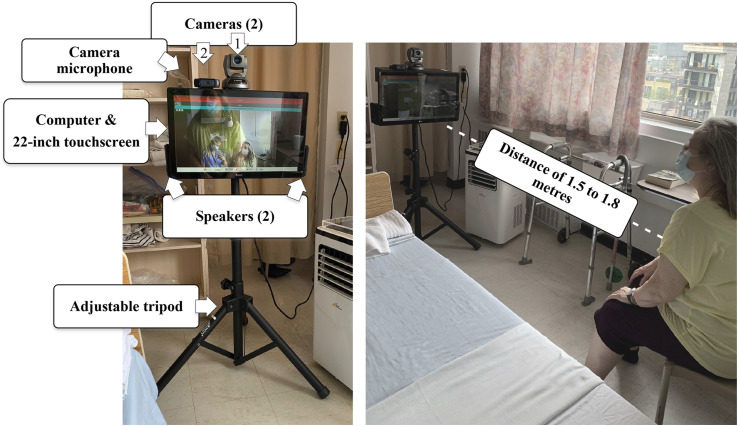
Telehealth kit and setup for the patient.

The portable telehealth kit had to be brought into the room by the PSWs at the beginning of each session. Each kit included a computer and a 22-inch touchscreen, two cameras (camera #1 was pan-tilt-zoom capable and remotely controllable to reframe the patient as needed, and camera #2 had a built-in microphone to capture the patient’s voice), as well as speakers on either side of the screen, all mounted on an adjustable tripod (see Figure 2). The kit was ideally placed about 1.5 to 1.8 metres from the patient to capture the person from head to toe in a seated position, with any potential obstacles removed to ensure patient safety.

During all sessions, one or more PSWs were around to ensure patient safety and communicate any necessary modifications to the dancing professional present online. In the event of communication problems using OpenTera+, the dance professionals and PSWs had each other’s cell phone numbers already pre-programmed into their phones so that they could communicate at any time during the live-stream sessions, if necessary. If an adverse event were to occur (a fall, fainting, sudden dizziness, or any other observable and concerning condition), the session was to be interrupted so a PSW could assist the participating patient and contact both the hospital’s neurological care coordinator and the therapist responsible for that patient. Such an event was then to be recorded in the research journal and the patient’s file. At the end of each session, the PSWs collected the kits and stored them in the designated area. The equipment was disinfected with diluted hydrogen peroxide wipes before and after use. Whether during or after sessions, the PSWs were invited to share any relevant information or observations, make recommendations, report problems, or relay patient comments. These follow-ups could be done by email, phone, or videoconference, at their convenience.

### Data collection and procedure

**Figure 3.**
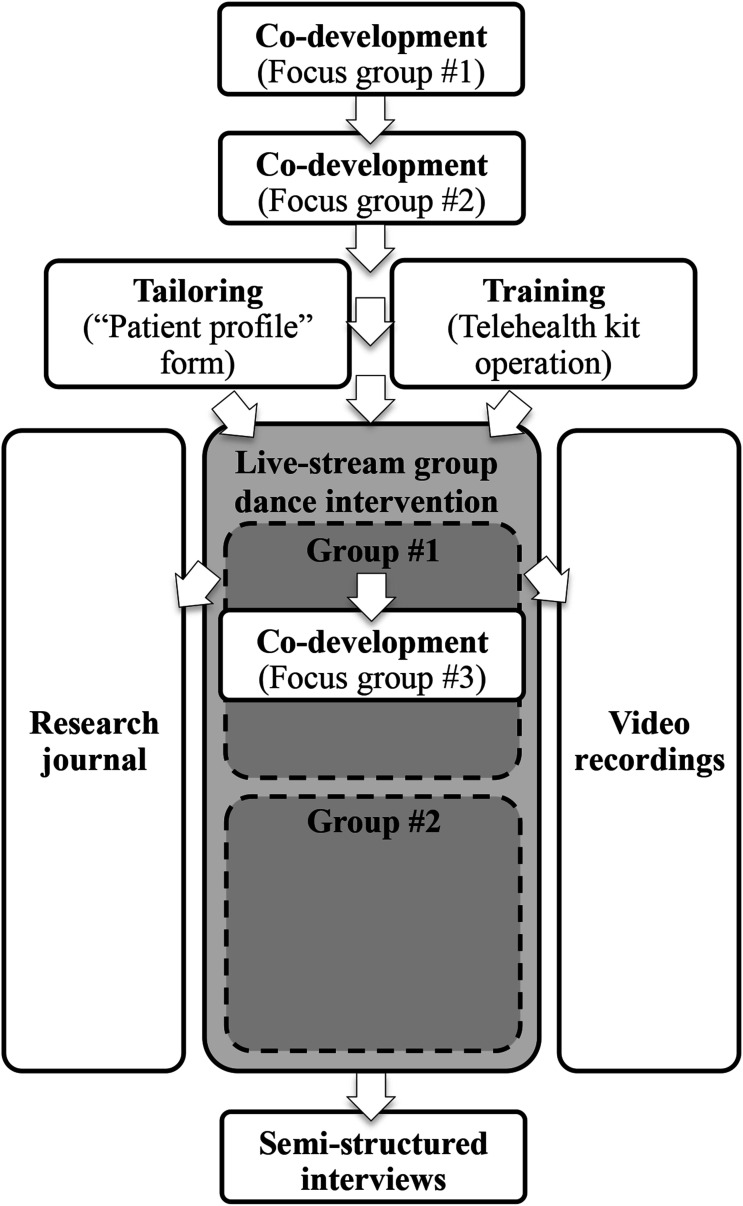
Data collection and procedure.

The feasibility study was intertwined with the co-development of the live-stream group dance intervention. For the co-development, focus groups brought together clinicians and dance professionals to discuss the rehabilitation needs to be considered in the intervention, and the rehabilitation principles to be integrated (see Figure 3). Led by the lead researcher, these focus groups were held using a set of guiding questions (see Table S2). Three 90-minute co-development meetings were held on a videoconferencing platform (Zoom Telecommunications Inc., San Jose, CA), two before the intervention to launch the co-development process and discuss a preliminary live-stream dance proposal, and one midway through the intervention with the first test group for adjustments. The meetings were video-recorded to produce a written summary of the ideas and comments made, sometimes accompanied by sketches to illustrate proposed movements.

**Figure 4.**
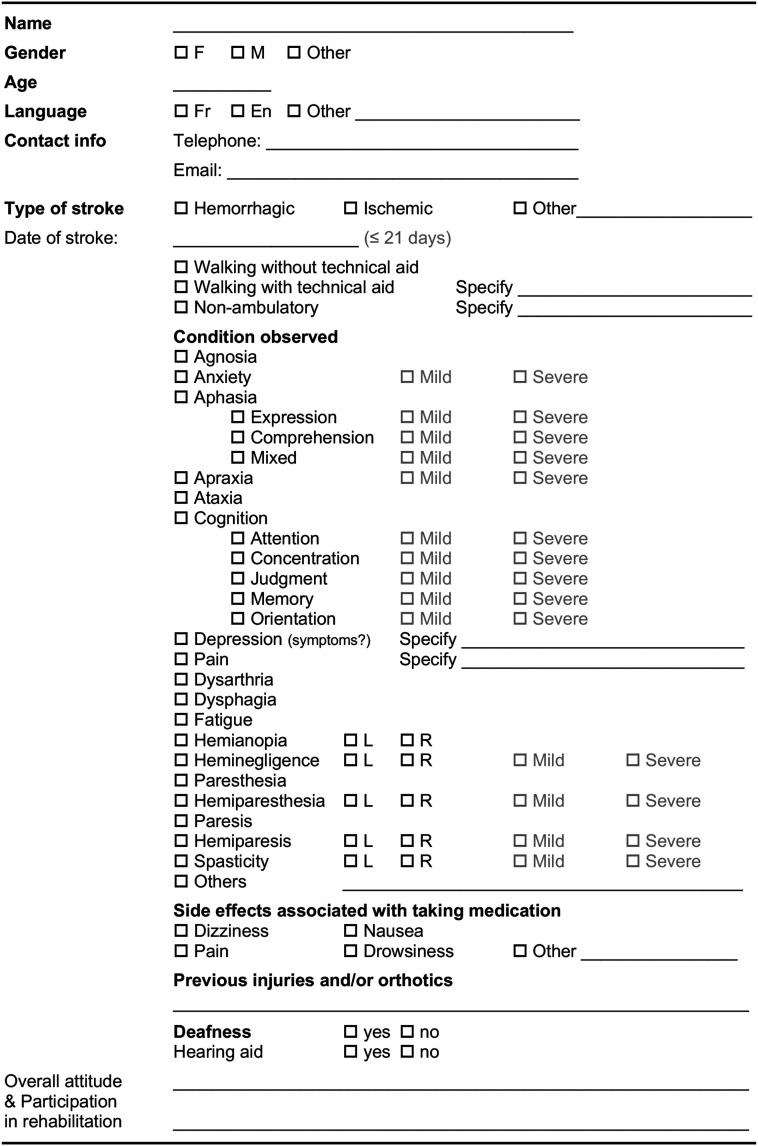
Patient profile form.

Before the start of the live-stream group dance intervention, medical information about each participating patient was collected from their medical records to complete a “patient profile” form (see Figure 4), with the neurological care coordinator’s collaboration. This information enabled the two dance professionals to adapt their dance content to the specific conditions of the patients participating in their group. A training session lasting approximately 30 minutes was held simultaneously for PSWs to explain how the portable telehealth kit works.

As soon as the live-stream group dance intervention began, the dance professionals compiled – in a research journal – observations and reflections relating to the medical, technical, and environmental factors that influenced the intervention. The follow-ups carried out by the PSWs were also recorded in the research journal. To enrich the journal notes and supplement them where possible, the live-stream dance sessions were recorded directly on the server and could be accessed by the research team with OpenTera+. At the end of the intervention, semi-structured interviews lasting approximately 30 minutes were conducted by telephone or with OpenTera, with both the patients and PSWs who had experienced the intervention. These interviews aimed to incorporate the patients’ and PSWs’ experiential perspectives into the feasibility study. The interviews were conducted by the lead researcher for the first group (sessions led by the research assistant), and by a research professional for the second group (sessions led by the lead researcher). The questions asked focused on the patients’ and PSWs’ experiences regarding the intervention (see Table S3), while seeking to document the medical, technical, and environmental factors that may have influenced their experience. The interview guide for PSWs included the same questions as for patients, but with slightly adapted wording. All interviews were audio-recorded and transcribed.

### Analysis

To co-develop the intervention’s dance content, the qualitative data were analyzed using analytical questioning,^33^ thus allowing a number of questions to be answered rigorously without resorting to thematization. The evolving investigative framework was anchored in the initial focus group guide aimed at operationalizing the co-development of the intervention (see Table S1). This framework evolved for the second and third focus groups to concentrate increasingly on dance content adaptation. The data thus underwent an iterative and continuous analysis process to allow for adjustments during the intervention.

Qualitative data collected through the research journal and semi-structured interviews underwent an inductive thematic analysis.^33^ The interviews transcripts were prepared by a research professional. The research assistant conducted a preliminary analysis of the notes and transcripts to make initial sense of the data and identify units of meaning, and thereby identify the themes likely to form the bases of the thematic coding tree. This initial preliminary analysis was discussed with the lead researcher in order to refine the coding framework, as there was no disagreement regarding the main themes identified. The lead researcher then proceeded to code all of the data collected. Next, the different themes were discussed with the research team until consensus was reached. All interviews were then coded and an analytical exercise was conducted to triangulate the data by unit of meaning, thus discriminating the perspectives of the different categories of participants. Data saturation was achieved with the recurrence of notes and comments collected. The video recordings were subjected to visual content analysis^33^ based on the themes identified through qualitative analysis. Cross-cutting thematic analysis was performed, and links were established between the main themes gleaned from the analysis. Finally, the average time required for telehealth kit management and patient assistance was calculated based on notes entered in the research journal, while the time required to manage technical problems was calculated based on the research journal and, in some cases, supplemented by video recordings.

## Ethical issues

The study received prior approval from the Human Research Ethics Committee of the Université du Québec à Montréal (4893_e_2021). As a private institution under contract with the provincial government, the partner site hosting the research did not have an ethics committee as such, but gave its consent after approval from the research suitability committee. The nature of the study was disclosed and explained to participants, and their consent was obtained before the study began. The data collected in this study were used solely for the purposes for which they were collected and within the context of this study. Unique codes were assigned to participant data and were stored on the secure cloud space of the Université du Québec à Montréal, to allow data analysis by the members of the research team. The data were processed anonymously by replacing participants’ names with identification numbers with no associated personal information. Participants were free to opt out of the study at any time with no consequences. The study was conducted in accordance with the ethical principles set forth in the Helsinki Declaration.

## Results

Seven clinicians (n=7), all women, responded to the invitation to participate in the co-development of the content for the live-stream group dance intervention, including physical therapists (n=4), an occupational therapist (n=1), a social worker (n=1), and a vascular neurologist (n=1). All the individuals approached to participate in the feasibility study accepted the invitation, namely six patients (n=6) and five PSWs (n=5). The patients included three women (group #1) and three men (group #2). The PSWs were all women. Two participated in the intervention with the first group, and one of the two participated in the intervention with the second group (the other three recruits did not take part in the research). The research team did not know any of the participants prior to the study. Given the numerous challenges encountered from the outset of the first group intervention, we carried out the project in two phases to provide pragmatic solutions. The results relating to the two phases are presented in the following sections, after those relating to the assistance procedure and dance content co-developed with clinicians.

### Assistance procedure and dance content

Regarding assistance procedure, analysis of data from the clinician focus groups revealed that assistance would be needed to manage the OpenTera interface and telehealth kit despite their simplicity, particularly because of the patient positioning required in relation to the telehealth kit (a distance of 1.5 to 1.8 metres). Moving towards or away from the kit could be difficult for some patients, so help from the PSW was deemed necessary for starting up and shutting down the telehealth kits and OpenTera platform. Assistance would also be needed to help patients optimize their participation, for example, by temporarily removing a tablet from the wheelchair or an orthosis when possible, to enable them to better perform the dance content. Assistance would therefore also be required at the end of the sessions to manage these technical aids.

In terms of dance content, two main safety-related concerns were identified prior to the actual dance content. Ensuring a safe intervention meant first limiting the risk of falling. All the dance movements to be developed therefore had to be performed sitting on a chair or wheelchair, with precautions taken to prevent any forward tilting. Safety concerns also required countering compensatory movements, with specific instructions aimed at minimizing certain potential movement patterns. After identifying the safety concerns, data analysis yielded six main categories of movement to be developed by the dance professionals to meet the specific needs of subacute post-stroke rehabilitation: 1) Body awareness, 2) Upper body movement, 3) Self-assisted movement, 4) Coordinated upper and lower body movement, 5) Breathing, and 6) Stretching. In the “body awareness” category, the movements aimed to stimulate the senses and foster body awareness by gently touching, rubbing, tapping, or massaging a part of the body. This type of movement involved gentle contact with the hands, arms, shoulders, torso, and thighs, as well as gentle body percussions (rhythmic expression using the body to create sounds and rhythms). These movements were accompanied by instructions that directed attention to the sensory-motor experience. As the intervention took place with the patient in seated position, the second movement category focused on “upper body movements”, i.e., movements involving the head and neck, entire spine, rib cage, shoulders, arms, and hands, including the pelvis and weight transfers from one buttock to the other. The movement usually started proximally (e.g., trunk) and then progressed toward the extremities (e.g., hands and fingers, or head). Through movement repetition, a greater degree of coordination could be introduced. “Self-assisted movements” were more specifically designed to stimulate the stroke-affected side of the body (especially the arm and hand – upper limb) with the assistance of the “strong” side. The movements developed consisted of using the “strong” arm and hand to assist the affected limb in movement (e.g., assisting the affected hand to slide it over a thigh). For the “coordinated upper and lower body movement” category, the movements were intended to integrate those previously explored with the upper body into a sequence that also involved the lower body in a coordinated manner. Leg movements were limited but still relevant to explore a more global experience of movement. Movements in the “breathing” category were designed both to remind patients to breathe while moving and to improve the quality of their breathing through movements that naturally encourage inhalation and exhalation (e.g., by straightening the spine or shortening the torso). Finally, the “stretching” movements were designed to provide gentle stretching, not only at the end of the session, but also sometimes at the beginning, to ensure that the body was better prepared for movement.

### Intervention with the first group – Phase 1 of the feasibility study

The first group consisted of three women (n=3) with a mean age of 71.3 years ± 8 (range 64-80), all recruited within ≤21 days of admission to the rehabilitation hospital. They had all suffered an ischemic stroke affecting the left hemisphere of the brain. They presented a wide range of post-stroke sequelae, including anxiety, fatigue, cognitive impairments, aphasia (expression and comprehension), speech articulation disorder, swallowing difficulty, mild or severe paralysis on one side of the body, and spasticity (see Table 1). Two PSWs (out of the five recruited) managed the portable telehealth kits and provided assistance to patients during sessions, when necessary. The sessions were led by one of the two dance professionals (DP1), live-streamed from a dance studio.

**Table 1.** Patient characteristics – Group #1.

Patient	Age	Gender	Stroke type & hemisphere lesion	Ambulation	Sequelae of the stroke
1	70	Female	Ischemic Left	Ambulatory (with a quad cane & orthosis)	Anxiety, dysarthria, dysphagia, paresthesia, paresis, hemiparesis
2	80	Female	Ischemic Left	Ambulatory (with a walker)	Fatigue, cognitive impairments (planning, problem solving), dysarthria, hemiparesis
3	64	Female	Ischemic Left	Ambulatory	Anxiety, mixed aphasia (mild for expression and comprehension), cognitive impairment (attention), dysarthria, hemiparesis, hemiplegia, spasticity

As reported in Table 2 summarizing feasibility outcomes, all patients and PSWs approached agreed to take part in the research and were recruited. A 75% adherence rate was observed for the first group. Data analysis identified three categories of factors that hindered the intervention, including participation. The first category involved medical factors (post-stroke fatigue and rehabilitation discharge); the second, technical factors (Internet and software connection issues), and the third, environmental factors (ambient noises and sound of music). The last two were significant enough to require adjustments before the intervention was carried out with the second group. In the meantime, they had to be dealt with as well as possible with the first group. For the PSWs, technical factors required a lot more time than anticipated; in addition to the time allocated for telehealth kit management and patient assistance at the beginning and end of sessions (including connecting to the software when starting the session and assisting a patient with removing and replacing her orthosis – an average of 36 extra min), time was also required during sessions to manage Internet and software connection issues (about 35 more minutes – see Table 2). Extra time was also required to manage ambient noises and the sound of music (another 8 min). The following subsections present in detail the three categories of factors that hindered the intervention.

**Table 2.** Feasibility outcomes – Group #1.

**Recruitment**					
Patient (approached)	(recruited)	PSW (approached)	(recruited)	Recruitment (%)	Justification for non-participation
3	3			100%	N/A
​		2	2	100%	N/A
**Adherence**					
Patient	Presence	Absence	Total number of sessions	Participation (%)	Justification for absences
1	6	2	8	75%	Internet connection issues
2	3	3	6	50%	Post-stroke fatigue, Rehabilitation discharge
3	8	0	8	100%	N/A
**Time required by psw**					
PSW	Setup, disinfection, & patient assistance		Internet & software connection issues	Ambient noises & sound of music	Patient assistance, storage, & disinfection
1 (patient #1)	∼ 10 min (orthotic assistance)		∼ 20 min	∼ 3 min	∼8 min
2 (patients #2-3)	∼ 10 min		∼ 15 min	∼ 5 min	∼8 min

### Medical factors – Post-stroke fatigue & rehabilitation discharge

Patient #2 dealt with severe post-stroke fatigue and missed half of the live-stream sessions. She felt particularly unwell (excessively tired) for one of the sessions. Each time, it was the PSW who went to meet her with the telehealth kit who pointed out the fatigue issue. Most of the time, the kit was still started up so that the dance professional could at least say hello to the patient, even if she was unable to dance with us that day. As this patient was discharged from the rehabilitation hospital after her sixth session, she was unable to complete the maximum potential of eight sessions.

### Technical factors – Internet and software connection issues

As detailed in Table 3, we identified two technical factors that hindered the intervention, the first being related to Internet connection issues. We had no choice but to use the public wireless network, which was not working reliably everywhere on the floors. For example, in patient #1’s room, the connection was particularly unstable due to its location (near a concrete wall); two of eight sessions were completely compromised for this participant, not to mention the inconvenience that this poor connection caused during the other sessions. Although this was particularly noticeable for patient #1, the other two patients also experienced an unstable Internet connection that sometimes disconnected them from the OpenTera platform, in turn requiring sustained PSW assistance. The dance professional had to call a PSW directly on her phone when a patient was alone at the time technical problems arose. The instability of the Internet connection also caused a lagging problem for the music, and mitigation strategies were necessary to limit the inconvenience caused by the audio lag. For example, we chose less rhythmic music (so that the lag would be less noticeable) and repeated instructions (to compensate for words that might have been lost in the lag).

**Table 3.** Technical and environmental factors.

**Technical factors – Internet connection issues**	
PSW2	“Sometimes, the start-up was slow. [I] don’t know if it was because the hospital’s connection wasn’t good. I didn’t understand where the problem was coming from.”
DP1	“[With the mute option activated], it became difficult to communicate with the patient. She could still hear me, but I couldn’t [hear her]. To resolve the Internet connection problem and refresh the connection, I had to contact the PSW by phone. (session 4)
**Technical factors – Software connection issues**	
DP1	“We still encounter some difficulties at the beginning of each session in getting the platform to detect users/patients and allow them in. [We really depend] on the help and tact of the PSWs, who have been proactive in assisting and resolving issues on site.” (session 5)
**Environmental factors – Ambient noises**	
P1	“[Y]ou know, there are people who are undergoing treatment, taking medication, and the nurse comes in with her machine and her stuff, bang bang, and she plugs it in and says, ‘Are you okay, sir?’; […] And then you [the dance professional] say, ‘Raise your arm up in the air, [lower it] to the side...’ [I]t’s difficult to get the full benefit of all the good things you offer us.”
P3	“It’s not the screen, sometimes it’s the environment it’s in. [T]here are lots of distracting elements around it. So, the screen loses its […] usefulness.”
P1	“Just a quiet space, you know... We can’t stop the hospital from running, but there are some quiet corners in the hospital. Perhaps we could have found another place.”
DP1	“[T]he sound is problematic in her room [patient #1] even though the PSWs are with her to support and recap [occasionally]. I feel that the challenges surrounding her are preventing her from achieving the level of autonomy she seems ready for.” (session 7)
DP1	“I feel frustrated with the fact that on either side of the screen, we are living opposite realities. I manage to get a super calm space, with participants muted, that leaves me with the illusion that all elements of the intervention (visual and auditory environment, pace and timing of my instructions, the choice of [movements], etc.) are working in symbiosis, whereas the reality for the three patients is different.” (session 4)
DP1	“The presence of a [PSW] during the session was important today since one signaled to me that one participant was feeling unwell and needed to stop and rest. Had we had not someone to help with relaying the message I would have not been able to detect that over distance and with the sound of her screen off.” (session 2)
**Environmental factors – Sound of music (for non-participating patients)**	
P1	Some [of my roommates] complained about the noise [music], some talked louder, some played music. It was distracting, and sometimes a little bit more complicated [to dance]. Why don’t we [patients] meet in a quiet room for our sessions?”
PSW1	“Since it was in the room, sometimes it was disturbing. [T]here were four of them [patients] in one room. So sometimes the sound [of music] bothered the others a little. And [patient #1] couldn’t hear very well, so we had to turn the volume up. After a while, [the other patients] understood that it was therapy and not just for fun, so it was okay. But we did get a few complaints at first.”

PSW = Personal support worker; DP = Dance professional; P = Patient.

Data sources: Semi-structured interview (PSW & P), Research journal (DP).

Streaming involves WebRTC technology, which is secure and used in all web browsers. The OpenTera+ application is built on the Qt platform, whose web component is integrated using the same technology as used by Google Chrome. More specifically, the STUN/TURN servers of WebRTC were hosted on our own servers to enhance security, with access control managed on a per-session basis. In this context, performance depended greatly on the quality of the participant’s Internet connection, i.e., the connection we could access within the rehabilitation hospital. The kits were connected via a wireless network, which could also have a significant impact on available bandwidth. However, WebRTC technology adjusts its bitrate and quality based on the available bandwidth to enhance the user experience.

The second technical factor hindering the intervention was related to problems connecting to the OpenTera software platform. PSW support was essential in dealing with these connection issues, mainly during the initial connection, but also during the session (once again, the location of patient #1’s room was particularly problematic). The platform would sometimes fail to immediately recognize the “patient profile” that had been created, requiring a restart. As mentioned earlier, the various technical problems required PSWs to spend much more time with patients than initially planned, resulting in disproportionate assistance needs.

### Environmental factors – Ambient noises & sound of music

Environmental factors, primarily ambient noises and the sound of music, were a major challenge during this first phase of the study. First, ambient noises in each room could be heard by the entire group through OpenTera (see Table 3). Since there were multiple sources of noise (e.g., visits from relatives, in-room therapies, room care, telephones, roommate’s television, etc.), we had to mute the microphones in the patients’ rooms and agree on some basic communication codes (thumbs up, thumbs down, raised hand) to ensure that everything was working properly for them (see Table 4). We opted for universal codes that everyone could quickly understand and apply, and dance professionals could always reactivate a patient’s microphone when necessary. Although this option helped minimize the impact of ambient noise on the OpenTera platform, it did not solve the noise problem experienced by the patients in their rooms, which was disruptive enough to interfere with their live-stream dance experience as they sometimes had difficulty hearing the instructions and music. At one point or another, all three patients expressed a desire to have their sessions somewhere other than in their rooms, which was impossible since no other space was available in the hospital. The patients’ experience was therefore quite different from that of the dance professional, who had control over her environment. During one session, the safety issue also arose when patient #1 felt a little unwell (excessively tired). Since her microphone was turned off and her greater-than-usual fatigue was difficult to detect on the dance professional’s screen, the patient was able to express her discomfort to the PSW. This isolated incident nevertheless confirmed the importance of always ensuring a communication channel for patient safety.

**Table 4.** Basic communication codes.

Thumbs up	Thumbs down	Raised hand
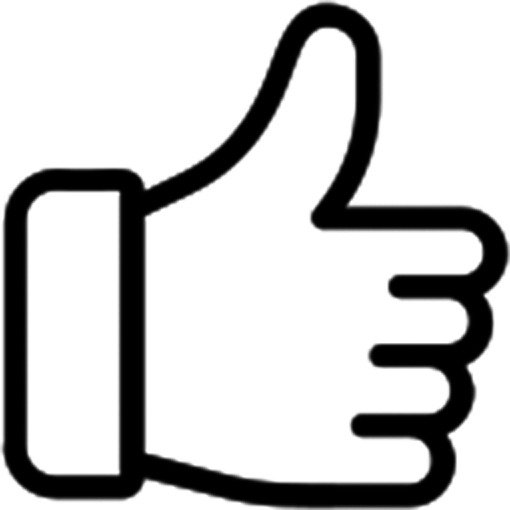	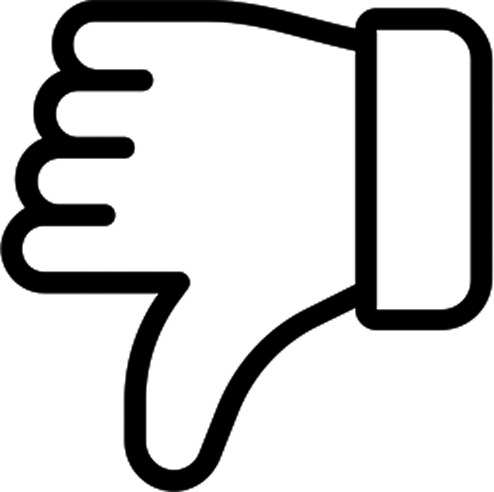	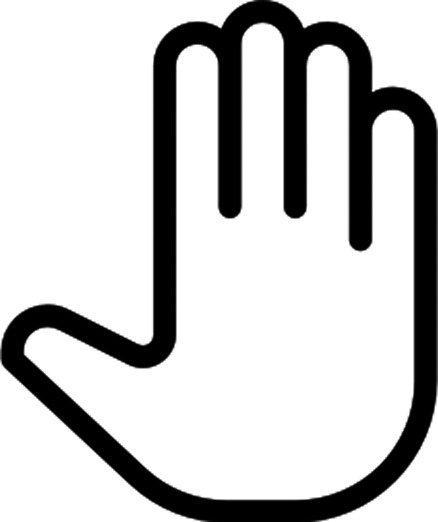
It is working well. No problem. I’m ready. I agree. I understood.	Something isn’t working. There is a problem. I am not ready.	Hold on a second. I would like to speak. I have a question.

The sound of the music used during the sessions also proved disturbing to other patients in the room at the same time as our participant. It turned out that all the patients recruited shared their rooms with other patients, which was not the scenario initially planned (combination of single-occupancy and shared rooms). We deliberately chose gentle, unobtrusive music to manage the problem as best we could, but it still irritated some roommates, at least until they understood that the intervention was part of the participating patients’ rehabilitation program. These musical choices were also made so as not to overload the attention of the participating patients who were already dealing with ambient noise. We assume that the dance professional’s voice also became irritating to patients who were not participating in the intervention, although this was not reported in the PSWs’ follow-ups or in the interviews.

### Intervention with the second group – Phase 2 of the feasibility study

The second group consisted of three men (n=3) with a mean age of 67 years ± 5.0 (range 62-72), all recruited within ≤21 days of admission to the rehabilitation hospital. All three had damage to the left hemisphere of the brain, two resulting from ischemic stroke and the third from hemorrhagic stroke. Like the first group, they presented a wide range of post-stroke sequelae including a speech articulation disorder, swallowing difficulty, mild or severe paralysis on one side of the body, endurance issues, motor control disorder, balance disorder while standing, memory impairment, pain, and headaches (see Table 5). One PSW took part in this second phase of the study, compared to two in phase 1. The live-stream dance sessions were led by the second dance professional (the lead researcher) from her office space (see Figure 1).

**Table 5.** Patient characteristics – Group #2.

Patient	Age	Gender	Stroke type & hemisphere lesion	Ambulation	Sequelae of the stroke
4	67	Male	Ischemic Left	Ambulatory (with a quad cane)	Dysarthria, dysphagia, hemiparesthesia, hemiparesis
5	72	Male	Ischemic Left	Ambulatory (with a cane or walker)	Dysarthria, dysphagia, endurance issue, motor control disorder, balance disorder (standing)
6	62	Male	Hemorrhagic Left	Ambulatory (with a half-walker)	Memory, dysarthria, paresis, endurance issue, balance disorder (standing), pain (left shoulder), headaches

Once again, as shown in Table 6 summarizing feasibility outcomes, all patients and PSWs approached agreed to take part in the research and were recruited. An 87% adherence rate was observed for this second group, which was deprived of one session out of the maximum of eight sessions planned, as the PSW responsible for the telehealth kits had to take a day off for health reasons. None of the other PSWs recruited were available. Patient #4 missed a session to undergo a medical examination and was discharged from rehabilitation services after the third session. Patient #6 was discharged after the sixth session.

**Table 6.** Feasibility outcomes – Group #2.

**Recruitment**					
Patient (approached)	(recruited)	PSW (approached)	(recruited)	Recruitment (%)	Justification for non-participation
3	3			100%	N/A
​		1	1	100%	N/A
**Adherence**					
Patient	Presence	Absence	Total number of sessions	Participation (%)	Justification for absences
4	2	1	3	66%	Medical exam
5	6	0	6	100%	N/A
6	7	0	7	100%	N/A
**Time required by psw**					
PSW	Setup, disinfection, & patient assistance		Internet & software connection issues	Ambient noises & sound of music	Patient assistance, storage, & disinfection
1 (patients #4-5-6)	∼ 15 min		N/A	N/A	∼10 min

Data analysis identified no factors that could have hindered the intervention (in particular, participation), firstly, because the medical condition of the group #2 participants did not include severe post-stroke fatigue (although the pain experienced by patient #6 had to be taken into account for some movements), and secondly, because solutions had been implemented to address the technical and environmental factors identified during the first phase. The following subsections present these solutions.

### Solution provided for technical factors – Connection to OpenTera and high-gain wireless

**Figure 5.**
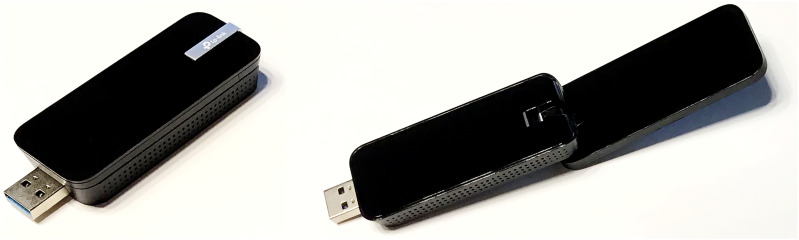
High-gain wireless USB adapter.

The problem connecting to the OpenTera software platform at the start of the sessions had been resolved by the software engineers before the second phase began, as it was a programming issue. The Internet connection problem was more troublesome. Since we still had to use the hospital’s public wireless network, we used high-gain wireless USB adapters (Archer T4U/AC1300 Mini Wireless MU-MIMO USB Adapter) plugged into the computers in our portable telehealth kits to improve the Internet connection (see Figure 5). These adapters stabilized the connection while also limiting the sound lag problem that affected voice and music in the first phase of the study.

By resolving the connection issue with the OpenTera software platform and improving the stability of our Internet connection, we managed to operate in the field with only one PSW during the second phase, and that person only provided assistance at the beginning and end of the sessions (with one exception). The average time required to ensure the smooth running of a session was 25 minutes for the three participating patients, compared to 79 minutes with the first group.

### Solution provided for environmental factors – Wireless noise-cancelling headphones

To manage both ambient noise in the rooms and the sound of music coming from our telehealth kits, we used Bose Noise Cancelling Headphones 700. This model of programmable noise-cancelling headphones (on a scale of 1 to 10) created a sound bubble for our participating patients without completely cutting them off from the world in case of an emergency (the sound was preset to 7 or 8). It met health criteria for infection prevention and control with a design covering the ear (rather than being inserted into the ear) and disinfectable materials (in terms of porosity). Each pair of headphones was associated with a specific patient and disinfected before and after use. Patients could talk to us through these headphones, thanks to their built-in microphone. This lightweight, adjustable wireless headphone model had a battery life of approximately 20 hours, meaning it did not need recharging during the month-long second phase of the study. Analysis of the interview data confirmed that the sound bubble provided by these headphones facilitated participation in the sessions and coexistence with hospital roommates. As patient #4 said, “It was fine, I was in a bubble.”

## Participants’ experiences of the live-stream group dance intervention – Phases 1 & 2

Apart from the factors that posed challenges for the intervention, the patients and PSWs recounted their experiences of the intervention during interviews, comments which we then cross-referenced with the notes taken by the two dance professionals in the research journal and with the video recordings. Three themes emerged from data analysis: “adapted dance content and pedagogy”, “engagement and reuse of dance content”, and “group effect and sense of belonging” (see Table 7). Regarding “adapted dance content and pedagogy”, the choice of movements echoing the exercises done during therapy and the way they were conveyed online via a computer screen proved important for the patients’ experience. Of the six main movement categories around which the intervention was developed beforehand, those relating to body awareness, breathing, and stretching stood out. The way dance was taught was also perceived as being well-suited to the patients’ condition and contributing to their positive experience. In terms of “engagement and reuse of dance content”, the enjoyable aspect of dance and its perceived complementarity to rehabilitation therapies fuelled engagement. Some patients even reused certain dance elements during their therapy sessions (especially breathing exercises), or even in their free time, for pleasure or because they found it relevant to add it to their therapy time. Finally, even though the sessions were held remotely, a “group effect and sense of belonging” was reported. It created a feeling of being with other people who had had a stroke and of being motivated by the participation and progress of others. Empathy and sensitivity were observed among the participants.

**Table 7.** Participants’ experience of the live-stream group dance intervention.

**Adapted dance content and pedagogy**	
P1	“[The dance professional] repeated, but repeated gently. [R]epetition was a good way to get me moving. [A]nd the dance, in a way, reused the exercises done in therapy.”
P2	“[T]he massage, the gentle movements, and the stretching—ultimately, it all became like relaxation. [A]nd by dancing, I could adapt my movements and be myself.”
P3	“Self-massage is something to discover and then try out. And in the end, stretching too. Of course, you can’t go as far [into movement] or as deeply, but gradually you become more and more flexible, and it becomes more and more beneficial.”
P4	“What I liked was the fact that [the dance professional] *really* looked at people through the screen. It allowed her to adapt to the patients ‘in front’ of her. You didn’t feel excluded when there were movements that required small adjustments.”
P5	“I liked that the movements were shown from the front on screen, and sometimes also from the side. Also, I could see myself on screen. All of this was very pedagogical.”
P6	“Conscious breathing... Breathing in, breathing out... In a way, these are simple movements, but they were so important. [A]nd dancing sometimes reminded me of what I did in therapy, but in a more organic way.”
PSW1	“What I liked was that it was adapted. If they couldn’t raise their hands, they put them on their thighs. They still did the movement, but with their hands on their thighs.”
**Engagement and reuse of dance content**	
P3	“Well, I’m glad I had your screen in my room, and that I heard about dancing, rather than a lot of other things! [laughs] And I could contribute to my recovery alone in my room, instead of wasting my time.”
P5	“The sessions were very positive because they were a pretty extraordinary complement... Because I was doing movements with my body, especially my shoulders, and they were difficult to do. […] I was able to do these movements, even when I was alone in my bed, I was doing them... I was doing them again and again.”
P6	“We did breathing exercises [in the dance sessions], and when I did this afterwards in my therapy sessions, at first, they thought I was tired! Well, no! I was trying to breathe while walking.”
PSW2	“The patients really liked it. You could see that they were looking forward to it... after the session, they felt good, they were eagerly awaiting it. [I]t increased the time spent in rehabilitation.”
PSW3	“I don’t know if she realized it or not, but it seemed like she [patient #1] was doing the movements that DP1 had taught her during the session. [S]he was repeating the same movements afterwards in her room... It really struck me, because I could see that she enjoyed it and was really getting into it.”
DP1	“She is a regular at the sessions and has not missed one class despite the difficult environment in her room, […] and her fatigue!” (session 5)
**Group effect and sense of belonging**	
P1	“Being with others... That was nice.”
P3	“It was my little group... Even from a distance, it was nice to be together.”
P6	“I liked seeing progress in others; it motivated me.”
PSW1	“[T]hey feel less alone. I think the fact that there are two or three other people creates a group effect. It makes them think, “Ah, I’m not alone.”
DP2	“It’s touching to see how empathetic the patients are towards each other. When one expresses a difficulty, I can feel the kindness in their eyes.” (session 4)

P= Patient; PSW = Personal support worker; DP = Dance professional.

Data sources: Semi-structured interview (P & PSW), Research journal (DP).

## Discussion

The live-stream group dance intervention successfully provided two additional hours of motor and cognitive stimulation per week during the second phase of the feasibility study, compared to significantly less time during the first phase due to various technical and environmental factors. Despite the significant impact of these factors on the dance experience, the patients still adhered to the proposed intervention.

The dance content co-developed upstream with clinicians was generally perceived by the patients as aligning with the exercises done during therapy, while the dance movements fostering body awareness, breathing, and stretching were particularly appreciated, judging from their feedback. The content aimed at improving body awareness through gentle “touching”, “rubbing”, “tapping”, and “massaging” offered a sort of adapted version of massage therapy for people with stroke in the form of self-massage, providing them with another form of tactile experience through physical touch.^34^ These movements, as well as those involving breathing and stretching, appeared to help relax the participating patients and ease their anxiety. Considering the challenges of stress and anxiety following a stroke,^35,36^ movements inspired specifically by massage deserve further investigation, especially since massage therapy has proven effective for stroke patients.^37,38^ However, such investigation should not exclude the other categories of dance-related movement (e.g., coordinated upper and lower body movement).

The first phase of the feasibility study highlighted several difficulties in conducting a live-stream dance intervention in the patients’ rooms, including issues related to ambient noise and cohabitation. We were therefore sensitive to the participating patients’ requests to access a quiet space or even a dedicated room that would bring the three patients together. However, this type of space was unavailable, thus confirming the documented difficulties regarding space availability.^14^ Even though live-stream dancing can foster connectivity,^39^ conceivably bringing the three patients together in the same place might have strengthened the group effect, despite the dance professional’s remote presence.^40^ This would likely pose a new challenge for the dance professional, that of clearly seeing each of the participants positioned at different angles relative to the computer camera.

The incident involving one participant who felt unwell confirmed unequivocally the importance of maintaining constant communication during this type of live-stream intervention. An eventual implementation of this intervention would require using as few human resources as possible on the ground to avoid increasing their workload (i.e., providing occasional assistance rather than sustained support), which underscores the importance of having a clear communication protocol for emergencies. In this instance, nothing in the patient’s nonverbal behaviour clearly indicated to the screen that she was feeling particularly unwell at any point. It can be difficult to detect certain non-verbal behaviours, such as subtle changes in muscle contractions that alter the appearance of facial features, in the focus of the gaze, or in body alignment.^41^ Furthermore, even with a good Internet connection and optimally controlled ambient noise, the person may have been unable to verbally express her discomfort. Therefore, two measures appear essential for the communication protocol: an emergency cell phone number allowing the dance professional to call a member of the on-site medical staff if needed at any time during a live-stream dance session (we had this in place), and an emergency button allowing the patient also to signal an emergency, if necessary (we had not put this in place). This safety concern also suggests that, in the subacute post-stroke rehabilitation context, it is wise to remain conservative and cautious by offering only seated dance movements unless the patient is always assisted.

By resolving the connection issue with the OpenTera telehealth software platform and significantly improving the stability of our Internet connection, we were able to operate in the field with only one PSW during the second phase; that person essentially provided assistance at the beginning and end of the sessions. The technical improvement was therefore significant, reducing assistance needs to a much more viable level. The location of our phase-2 participants’ rooms seemed less problematic in terms of connectivity, as they were further from the concrete walls. The proposed solution might therefore not have yielded such good results in other rooms.

The telehealth kits were portable but still had a non-negligible weight. Although the PSWs did not complain about them during the interviews, these kits were also bulky and sometimes difficult to store in dedicated spaces. Such obstacles were manageable for a pilot study, but all the necessary equipment should ideally be accessible in the patients’ rooms to avoid having to transport it in and out of the rooms. This would limit PSW handling of equipment and provide an alternative to using adjustable tripods, which were cumbersome in the limited room space. Another alternative would be to use electronic tablets (such as iPads), which are more portable and easier to store. That said, not all hospitals are equipped to support virtual care/services in rooms, which brings us back to the potential use of a common room where small groups could gather for their live-stream dance sessions. Such a solution would require a minimum of equipment. It then remains to be seen whether having a professional dance instructor come to the hospital for in-person sessions might be preferable, if the space provided allows for an in-person group intervention.

Co-developed with clinicians, this intervention was intended to be adapted for a stroke clientele to offer complementary therapy in settings with sometimes very little space available for group activities. In a context where programs focus particularly on individualized rehabilitation,^42^ our live-stream dance intervention could not, however, be that individualized and specific. Ideally, such group sessions could be supplemented by individual ones with patients who wish to participate, allowing for more targeted dance adaptations tailored to their rehabilitation program. That said, we observed that, although not highly individualized, our dance intervention “harnessed humanity” in rehabilitation exercises.^43^

### Strengths and limitations

With its inclusive selection of patients, this feasibility study sought to be as representative as possible of the experience that can be generated by a live-stream group dance intervention in the context of subacute post-stroke rehabilitation, with all the challenges that heterogeneous profiles entail. The co-development of the dance content with clinicians contributed to the safety and relevance of the intervention prior to the feasibility study; ideally, patient partners should also have been involved, but that was impossible for this study. The semi-structured interviews were conducted by a research assistant and research professional unknown to the participants to limit social desirability bias. The dance professionals who delivered the intervention were both involved in data analysis, but a qualitative analysis process and peer debriefing were used to ensure rigour in coding. The findings of this study cannot be generalized as it was a qualitative study with a very limited number of participants. Despite the small study sample, the findings provide relevant information for developing and implementing live-stream dance interventions in the context of subacute post-stroke rehabilitation. While data saturation was enhanced by obtaining diverse perspectives (patients’ and PSWs’, through interviews; dance professionals’, through the research journal), different participation experiences could emerge from other types of dance interventions, other dance professionals, or other participant groups.

## Conclusion

A live-stream group dance intervention in subacute post-stroke rehabilitation appears feasible as a means of intensifying rehabilitation (adding therapy hours). Dance sessions can be offered in a safe and complementary manner to stimulate small groups of people simultaneously through dance. In our study, movement categories such as “body awareness”, breathing”, and “stretching” were particularly appreciated and sometimes reused in regular therapies, or even during personal time. Motivation and engagement were observed in most participating patients. That said, such an intervention poses several environmental and technical challenges that must be overcome to provide participating patients with a quality experience. Wearing noise-cancelling headphones proved useful for controlling ambient noise in the patients’ hospital rooms and for respecting hospital roommates who were not participating in the intervention. The use of devices such as high-gain wireless adapters should be considered to optimize the connection to a public Internet network within a rehabilitation hospital, or the use of a private or more stable wireless network when available. For an even more personalized dance experience, this type of group dance intervention could be combined with individual “tailor-made” sessions for more personalized adaptation of dance content.

## Supplemental material

Supplemental material - Feasibility of a live-stream group dance intervention with inpatients in subacute post-stroke rehabilitation: A pilot study Supplemental material for Feasibility of a live-stream group dance intervention with inpatients in subacute post-stroke rehabilitation: A pilot study by Lucie Beaudry , Corinne Skaff, Simon Brière, Dominic Létourneau , Michel Tousignant , François Michaud in DIGITAL HEALTH

Supplemental material - Feasibility of a live-stream group dance intervention with inpatients in subacute post-stroke rehabilitation: A pilot study Supplemental material for Feasibility of a live-stream group dance intervention with inpatients in subacute post-stroke rehabilitation: A pilot study by Lucie Beaudry , Corinne Skaff, Simon Brière, Dominic Létourneau , Michel Tousignant , François Michaud in DIGITAL HEALTH

Supplemental material - Feasibility of a live-stream group dance intervention with inpatients in subacute post-stroke rehabilitation: A pilot study Supplemental material for Feasibility of a live-stream group dance intervention with inpatients in subacute post-stroke rehabilitation: A pilot study by Lucie Beaudry , Corinne Skaff, Simon Brière, Dominic Létourneau , Michel Tousignant , François Michaud in DIGITAL HEALTH
