# Supplementary material for: Feasibility of a live-stream group dance intervention with inpatients in subacute post-stroke rehabilitation: A pilot study
Source: Digit Health. 2026 Jun 5;12:20552076261459521. doi: 10.1177/20552076261459521 (PMC13241686; doi:10.1177/20552076261459521)
Supplement: Supplemental material - Feasibility of a live-stream group dance intervention with inpatients in subacute post-stroke rehabilitation: A pilot study [file sj-pdf-1-dhj-10.1177_20552076261459521.pdf]

Table S1. COREQ (COnsolidated criteria for REporting Qualitative research) checklist*

**Topic**	**Item No.**	**Guide Questions/Description**	**Reported on Page No**
**Domain 1: Research team and reflexivity**			
Personal characteristics			
Interviewer/facilitator	1	Which author/s conducted the interview or focus group?	6 – 7
Credentials	2	What were the researcher's credentials? e.g. PhD, MD	3 – 4
Occupation	3	What was their occupation at the time of the study?	3 – 4
Gender	4	Was the researcher male or female?	3 – 5
Experience and training	5	What experience or training did the researcher have?	3 – 4
Relationship with participants			
Relationship established	6	Was a relationship established prior to study commencement?	8
Participant knowledge of the interviewer	7	What did the participants know about the researcher? (e.g. personal goals, reasons for doing the research)	4
Interviewer characteristics	8	What characteristics were reported about the interviewer/facilitator? (e.g. bias, assumptions, reasons and interests in the research topic)	6 – 7, 16
**Domain 2: Study design**			
Theoretical framework			
Methodological orientation and Theory	9	What methodological orientation was stated to underpin the study? (e.g. grounded theory, discourse analysis, ethnography, phenomenology, content analysis)	3
Participant selection			
Sampling	10	How were participants selected? (e.g. purposive, convenience, consecutive, snowball)	4
Method of approach	11	How were participants approached? (e.g. face-to-face, telephone, mail, email)	4 – 5
Sample size	12	How many participants were in the study?	8
Non-participation	13	How many people refused to participate or dropped out? Reasons?	8
Setting			
Setting of data collection	14	Where was the data collected? (e.g. home, clinic, workplace)	6 – 7
Presence of non-participants	15	Was anyone else present besides the participants and researchers?	No
Description of sample	16	What are the important characteristics of the sample? (e.g. demographic data, date)	4, 9 – 10, 12, Tables 1 & 5
Data collection			
Interview guide	17	Were questions, prompts, guides provided by the authors? Was it pilot tested?	Tables S2 & S3
Repeat interviews	18	Were repeat interviews carried out? If yes, how many?	No
Audio/visual recording	19	Did the research use audio or visual recording to collect the data?	6 – 7
Field notes	20	Were field notes made during and/or after the interview or focus group?	6 – 7
Duration	21	What was the duration of the interviews or focus group?	6 – 7
Data saturation	22	Was data saturation discussed?	7 – 8, 16
Transcripts returned	23	Were transcripts returned to participants for comment and/or correction?	No
**Domain 3: analysis and findings**			
Data analysis			
Number of data coders	24	How many data coders coded the data?	7
Description of the coding tree	25	Did authors provide a description of the coding tree?	No

Derivation of themes	26	Were themes identified in advance or derived from the data?	7 – 8
Software	27	What software, if applicable, was used to manage the data?	N/A
Participant checking	28	Did participants provide feedback on the findings?	No
Reporting			
Quotations presented	29	Were participant quotations presented to illustrate the themes/findings? Was each quotation identified? e.g. participant number?	13, Tables 3 & 7
Data and findings consistent	30	Was there consistency between the data presented and the findings?	8 – 14
Clarity of major themes	31	Were major themes clearly presented in the findings?	8 – 14
Clarity of minor themes	32	Is there a description of diverse cases or discussion of minor themes?	10, 15

* Developed from: Tong A, Sainsbury P, and Craig J. Consolidated criteria for reporting qualitative research (COREQ): A 32-item checklist for interviews and focus groups. *Int J Qual Health Care* 2007; 19: 349–357.
