# Supplementary material for: Feasibility of a live-stream group dance intervention with inpatients in subacute post-stroke rehabilitation: A pilot study
Source: Digit Health. 2026 Jun 5;12:20552076261459521. doi: 10.1177/20552076261459521 (PMC13241686; doi:10.1177/20552076261459521)
Supplement: Supplemental material - Feasibility of a live-stream group dance intervention with inpatients in subacute post-stroke rehabilitation: A pilot study [file sj-pdf-2-dhj-10.1177_20552076261459521.pdf]

Table S2. Focus group guide (with clinicians)

Key questions	Complementary questions	Clarification questions
Prioritized needs and intervention targets		
For stroke rehabilitation patients, what should a live-stream group dance intervention focus on?	What needs should be considered? (What would you recommend in this regard?)<br>How could such an intervention complement therapy?<br>What should we focus on in dance? (What dimensions/characteristics?)	Can you tell me a little more about that?<br><br>Can you give me an example?
Security and assistance needs		
What risks and limitations do you anticipate for a live-stream group dance intervention conducted remotely?	What limitations/problems can be anticipated?<br>What should be anticipated in terms of skills? Technology-wise?	Can you tell me a little more about that?<br><br>Can you give me an example?
Based on this list, what are some potential solutions?	What should be monitored, limited, or countered?<br>(Review the anticipated risks and associate them with a solution—including adapting the dance or monitoring procedures).	
What kind of assistance will patients need?	How can we make dancing easier?<br>What kind of technological support would you like to see?	
Telerehabilitation practices – examples and inspiration		
What examples/inspiration could be applied to a live-stream dance intervention?	Are there any strategies worth mentioning as examples from other disciplines? Any methods?	Can you tell me a little more about that?
**Other**	Before concluding, is there anything else you would like to add regarding the development of the intervention?	
