# Supplementary material for: Feasibility of a live-stream group dance intervention with inpatients in subacute post-stroke rehabilitation: A pilot study
Source: Digit Health. 2026 Jun 5;12:20552076261459521. doi: 10.1177/20552076261459521 (PMC13241686; doi:10.1177/20552076261459521)
Supplement: Supplemental material - Feasibility of a live-stream group dance intervention with inpatients in subacute post-stroke rehabilitation: A pilot study [file sj-pdf-3-dhj-10.1177_20552076261459521.pdf]

Table S3. Semi-structured interview guide (with patient)

**Experience of the live-stream dance intervention**	
	**1.** How did you experience the live-stream dance intervention? Tell me about it...<br>- What did you appreciate the most?<br>- What did you like least? Or find difficult?<br>- Are there any moments that you remember in particular? Which ones?<br><br>**2.** What did you think of dancing via a computer screen?<br>- Did you encounter any difficulties participating on your own? What kind of help did you need?<br><br>**3.** Tell me what made it easier for you to participate? What made it difficult?
**Other**	**4.** Is there anything else you would like to tell me before we finish?
